# Adaptive Neuro-Fuzzy Inference System and a Multilayer Perceptron Model Trained with Grey Wolf Optimizer for Predicting Solar Diffuse Fraction

**DOI:** 10.3390/e22111192

**Published:** 2020-10-22

**Authors:** Randall Claywell, Laszlo Nadai, Imre Felde, Sina Ardabili, Amirhosein Mosavi

**Affiliations:** 1 Kando Kalman Faculty of Electrical Engineering, Obuda University, 1034 Budapest, Hungary; randal.claywell@uni-obuda.hu (R.C.); nadai@uni-obuda.hu (L.N.); s.ardabili@ieee.org (S.A.); 2 John von Neumann Faculty of Informatics, Obuda University, 1034 Budapest, Hungary; felde@uni-obuda.hu; 3 Environmental Quality, Atmospheric Science and Climate Change Research Group, Ton Duc Thang University, Ho Chi Minh City, Vietnam; 4 Faculty of Environment and Labour Safety, Ton Duc Thang University, Ho Chi Minh City, Vietnam

**Keywords:** machine learning, prediction, adaptive neuro-fuzzy inference system, adaptive network-based fuzzy inference system, diffuse fraction, multilayer perceptron (MLP), renewable energy, solar energy, photovoltaics, data science, solar irradiance, big data, solar radiation

## Abstract

The accurate prediction of the solar diffuse fraction (DF), sometimes called the diffuse ratio, is an important topic for solar energy research. In the present study, the current state of Diffuse irradiance research is discussed and then three robust, machine learning (ML) models are examined using a large dataset (almost eight years) of hourly readings from Almeria, Spain. The ML models used herein, are a hybrid adaptive network-based fuzzy inference system (ANFIS), a single multi-layer perceptron (MLP) and a hybrid multi-layer perceptron grey wolf optimizer (MLP-GWO). These models were evaluated for their predictive precision, using various solar and DF irradiance data, from Spain. The results were then evaluated using frequently used evaluation criteria, the mean absolute error (MAE), mean error (ME) and the root mean square error (RMSE). The results showed that the MLP-GWO model, followed by the ANFIS model, provided a higher performance in both the training and the testing procedures.

## 1. Introduction

Estimation of solar irradiance is of utmost importance for the efficient operation of solar energy production operations [1]. Insight into the solar irradiance levels are beneficial to managing solar facilities and passive energy-efficient systems [2]. The value of global irradiance consists of direct and diffuse solar irradiance and the ratio that exists between. The direct and diffuse solar irradiance are essential for estimating solar irradiance under arbitrary surface orientations [3,4], obstructed environments [5], within interior spaces [6], for building energy simulations, impact on photovoltaic systems and the photosynthesis potentials in agricultural/forestry analysis and planning [7,8]. Recent studies have shown and measured the positive effect that diffuse irradiation has on increasing canopy light use efficiency (LUE) in the Amazon rain forest and related vegetative carbon uptake [9,10,11].

Solar irradiance varies greatly with latitude, surface inclination, terrain, season and time (with different, but predictable solar positions) and is subject to unpredictable weather conditions [12]. Many models have been evaluated for their ability to predict the diffuse fraction with varying degrees of success [13]. One study statistically compared nine models for estimating the diffuse fraction, using 10 years (1996–2005), of hourly global and diffuse solar radiation data and only identified three models for further evaluation [14]. Another study considered ten models for hourly diffuse irradiation and evaluated their performance, both in their original and locally adjusted versions, against data recorded at five sites from a subtropical-temperate zone in the southern part of South America (latitudes between 30° S and 35° S). The best estimates resulted from locally adjusted multiple-predictor models, some of which can estimate the hourly diffuse fraction with an uncertainty, of 18% of the mean [15]. In general, most researchers agree that low solar altitudes and a low clearness index (cloudy conditions), cause problems for diffuse fraction empirical modeling and most models are site-dependent [16,17].

According to the international energy agency (IEA), Renewable electricity capacity is expected to grow by over 1 TW, a 46% growth, from 2018 to 2023. Photovoltaic (PV) accounts for more than half of this expansion (575GW). This growth will accelerate from 2020 onward and will be driven by supportive government policies and market improvements across most regions [18]. Solar PV generates power from sunlight, transforming solar irradiance into power. PV system performance is directly affected by uncertain weather conditions (cloud cover, temperature, pollution, time-of-year). This creates challenges in PV electrical generation and power output predictions.

There are existing solutions for addressing these challenges, such as, battery storage and heat storage, that can compensate for irregular PV and power production. In addition, if one could estimate how much PV power can, theoretically, be produced within a given timescale (hourly, daily, weekly, monthly), the operational costs for solar power facilities could be significantly reduced. Therefore, accurate solar irradiance forecasting is critical for the efficient production of a solar-related, electrical supply, in a local grid. Since PV power output is dependent on solar irradiance, solar irradiance forecasting has been a hot topic of research in the literature. Forecasting methods can be split into three basic methodologies, i.e., physical models, statistical models, and machine learning (ML) models. 

The prediction of a solar economy, for a given location, is not only important for power forecasting, but also, for energy-efficient buildings. These methodologies can encompass one or incorporate a combination all three of the above methodologies. Using graphical/statistical predictive methods has been around for a long time. In 1993, National Renewable Energy Laboratory (NREL) presented a quality control, computer mapping system that illustrated the qualities of a regions solar economy and allowed for visually identifying outliers [19]. It took advantage of two dimensionless solar quantities called the “diffuse fraction” and the “clearness index”. There has been work done involving the use of this graphic tool involving statistical methods to develop statistically superior “quality envelopes” to identify errors in solar data and map/predict a regions solar potential [20]. More recently, this graphic/statistical methodology has been fine-tuned by using the diffuse fraction, k_d_, and the clearness, k_t_, to provide the possibility of a new approach to solar radiation decomposition and the diffuse fraction, founded on physical-based correlations [9,21,22].

Physical models make predictions based on the physical characteristics that manifest themselves in weather. Statistical models are based on historical/time-series data and are more basic than the Physical models, they are often limited by assumptions based on normality, linearity and/or certain variable dependencies. However, ML models can discover and acquire the non-linear relationships between input and output data, without being explicitly programmed for the task [22,23]. The larger the volume of data and depth of the dataset offers the potential of a very accurate diffuse fraction prediction. Jamil and Akhtar (2017) have preliminarily performed a study in Southern India, involving standard pyranometers and a continuous data collection facility, to acquire solar data for three years in Aligarh’s city (27.89° N, 78.08° E). The related dataset was divided into two parts involving a ‘training dataset’ to develop the models, while a ‘validation dataset’ was used to test the models. Reasonable agreement was found between the model estimates and the measured data [24]. Tapakis et al. (2016) employed an artificial neural network (ANN) for the prediction of solar DF in the presence of global irradiance on the horizontal plane, extraterrestrial irradiance on the horizontal plane and the time of the day as the independent variables. Results had been analyzed using root mean square error (RMSE) and determination coefficient values. According to the results, ANN could successfully cope with the prediction task with a high accuracy [25]. Lauret et al. (2014) employed the Bayesian statistical approach to develop the hourly solar DF model. Results have been evaluated by mean bias error (MBE) and RMSE values. According to the results, the developed model could successfully overtake the classical statistical models in the term of accuracy [26]. Elminir et al. (2007) developed ANN model for the prediction of solar DF. The developed model employed the clearness index and sunshine fraction as the independent variables. According to the results, ANN could successfully cope with the prediction task [27]. Rehman and Mohandes (2007) developed an ANN-based model for the prediction of solar DF. Day of the year and daily mean air temperature have been considered as the independent variables for the prediction of solar DF. Results have been evaluated by the use of mean square error (MSE) values. According to the results, the relative humidity along with daily mean temperature as the input variables of the ANN could successfully reduce the MSE value. As is clear, ANN can be considered the promising single ML-based model for solar DF prediction. Accordingly, it has been decided to employ two hybrid ANN-based models for the prediction of the solar DF in the present study. Therefore, three ML-based models will be considered in this work. Specifically, a single MLP, a hybrid ANFIS, and finally, a hybrid MLP-GWO, will be evaluated for prediction performance using various irradiance data from Almeria, Spain over a period of almost eight years. Section 2 describes the data and methods used and a detailed description of the three models with the error evaluation metrics (MAE and RMSE) used. Section 3 provides the results of the performance of the two models, an error analysis and comparison data. Section 4 is a short description of current work in the area of diffuse irradiance prediction and a discussion of the process. Finally, Section 5 presents conclusions and future work.

## 2. Data and Methods

### 2.1. Data 

The data used herein, was measured in Almería (Spain), from a horizontal rooftop, located at the University of Almería (36.83° N, 2.41° W and 680 AMSL). Almería is in a Mediterranean Coastal Area, in the South-eastern region of Spain. This location has a high frequency of cloudless days, an average annual temperature of 17 degrees celsius, and a high humidity environment, as would be expected near the sea [28]. The global and diffuse irradiance data were collected via Kipp and Zonen (Model-CM11) pyranometers. One unit had an Eppley (model SBS) shadow-band fixed, to measure the diffuse irradiance. The beam normal irradiance was measured using an Eppley normal incident pyrheliometer (Model-NIP). The original data set consisted of daily sunrise to sunset hourly values centered on GMT of measured global and diffuse horizontal irradiance, and beam normal irradiance readings, were observed over a period of 2829 days (1 June 1990 through 28 February 1998). The entire data set contained 12,435 of daylight records. The data was quality-controlled and marked for missing time-stamps, equipment/power malfunctions and other erroneous readings. The data used for input/output/validation was the solar-related data, found in Table 1, the dataset had other metrological readings, such as, relative humidity, etc., these data were not used.

### 2.2. Normalization

Normalization was performed due to the differences in the parameters range. Equation (1) presents the formula which normalizes the parameters between −1 to +1. Accordingly, the formula employs the minimum and maximum values and produces normalized values between −1 and +1. This process can reduce errors that arise from large differences in the parameters range. Equation (1) was extracted from [29] as:

(1) $$x_{N}\;= [\left(\frac{x-X_{min}}{X_{max}-X_{min}}\right)\times 2]-1$$

where, $x_{N}$ is the normalized data, $X_{min}$ is the lowest number and $X_{max}$ is the highest number in the dataset.

### 2.3. Methods

#### 2.3.1. Multi-Layered Perceptron (MLP)

**Figure 1:**
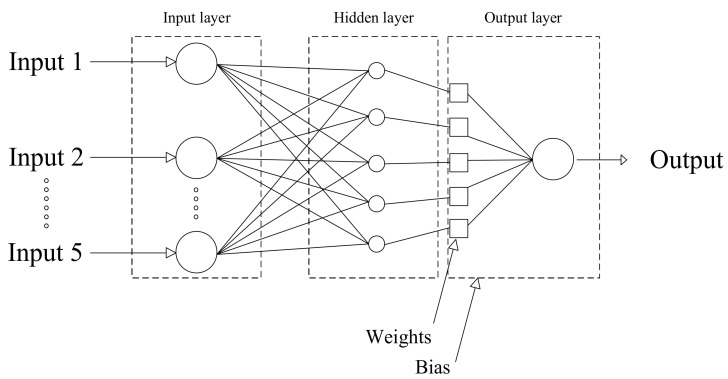
Architecture of the multi-layer perceptron (MLP) model.

MLP as a feed-forward ANN method, can successfully generate the values of the output variables, according to the input variables, through a non-linear function. A simple architecture of an MLP model is represented in Figure 1. According to Figure 1, the MLP contains three main sections. First, section imports input variables, the second section is called as the hidden layer and includes set of neurons which are called the neurons in the hidden layer. The number of neurons in the hidden layer are one of the adjustable factors that can affect the accuracy of the MLP model. The final layer is called the output layer and it contains the output variables [30]. Figure 1 also echoes the architecture of the MLP model, adopted from [31]. 

This model has been frequently cited in various studies. The present section only mentions the main and important aspects, concerning MLP. 

In a MLP, a hidden layer connects the input layer to the output layer and produces the output value (f(x)) using Equation (1), below [32]:

(2) $${f:R}^{I}\to R^{O}\;f\left(x\right){=K(b}^{\left(2\right)}{+w}^{\left(2\right)}\left(Q\left(b^{\left(1\right)}{+w}^{\left(1\right)}x\right)\right))$$

where, K and Q refer to the activation functions and b and w refer to the bias and weights, respectively. A hidden layer can be introduced by Equation (2) [32]:

(3) $$h\left(x\right){=Q(b}^{\left(1\right)}{+w}^{\left(1\right)}x)$$

According to [32] the two common activation functions for Q can be represented by Equations (3) and (4).

(4) $$\mathrm{Tanh}\left(x\right){=(e}^{x}{+e}^{-x}{)/(e}^{x}{-e}^{-x})$$

(5) $$\mathrm{Sigmoid}\left(x\right){=1/(1+e}^{-x})$$

Tanh(x) can do the task faster than Sigmoid(x). The output vector according to [32] can be calculated by Equation (5).

(6) $$o\left(x\right){=K(b}^{\left(2\right)}{+w}^{\left(2\right)}h\left(x\right))$$

In the present study, the architecture of the MLP has one input layer including five solar inputs:

- Global irradiance;
- Beam normal irradiance;
- Sunshine index;
- k_t_ (clearance index–global/extraterrestrial);
- k (diffuse/extraterrestrial).

There was one hidden layer including 15, 20, 25 and 30 neurons in the hidden layer, for finding the optimum number of neurons in the hidden layer and one output layer, including one output, the Diffuse Fraction **k_d_**. The activation function was selected to be the Tanh type. Training was performed by 80% of the total data. Training was started with 15 neurons, with three repetitions for finding the best run, due to the change in the results of the MLP in each training and the instability of the results in each repetition. This section seeks to provide the best architecture for the MLP to be optimized, by the grey wolf optimizer (GWO) method, discussed in the next section. 

#### 2.3.2. MLP-GWO

The GWO is known as a metaheuristic algorithm, which is implemented mimicking the social behavior of grey wolves, while hunting, in the wild. In fact, in the process of finding the best solution for the cost function, is considered as the prey and the hunting in the process, as the wolves move towards prey with a their unique hunting strategy. The accuracy of the algorithm depends on the population of the wolves [33]. During the hunt, grey wolves surround the prey. The following equations describe the mathematical models, where: t refers to the current iteration, A and C refer to coefficient vectors, X_p_ refers to the prey position vector, and X refers to the grey wolf position vector.

(7) $$\vec{d}=\left|{\vec{c}\times x}_{p}(t)-\vec{x}(t)\right|$$

(8) $$\vec{x}\left(t+1\right)=x_{p}\left(t\right)-(2\vec{a}\times \vec{r_{1}}-\vec{a})\times \vec{d}$$

(9) $$\vec{c}=2\times \vec{r_{2}}$$

In the above relationships, the variable a, decreases linearly from 2 to 0 during the iterations, and r_1_, r_2_ are random vectors in the range [0, 1]. Hunting operations are usually led by alpha, beta and delta wolves, may occasionally hunt. In the mathematical model of grey wolf hunting behaviors, we assume that alpha, beta, and delta have better knowledge of the potential prey position. The first three solutions are best stored and the other agent is required to update their positions, according to the position of the best search agents, as illustrated in the following equations.

(10) $$\vec{d_{\alpha }}=\left|\vec{c_{1}}{\times x}_{\alpha })t(-\vec{x}(t)\right|;\;\vec{d_{\beta }}=\left|\vec{c_{2}}{\times x}_{\beta }\left(t)-\vec{x}(t\right)\right|;\;\vec{d_{\delta }}=\left|\vec{c_{3}}{\times x}_{\delta }\left(t)-\vec{x}(t\right)\right|$$

(11) $$\vec{x}\left(t+1\right)\;=\;\frac{\vec{x_{1}}+\vec{x_{2}}+\vec{x_{3}}}{3}$$

The main algorithm of the GWO can be characterized as follows [33,34].

1. The fitness of all solutions are calculated and the top three solutions are selected as alpha, beta and delta wolves until the algorithm is finished.
2. In each iteration, the top three solutions (alpha, beta and delta wolves) are able to estimate the hunting position and do so, in each iteration.
3. In each iteration, after determining the position of alpha, beta and delta wolves, the position of the rest of the solutions are updated by following them. During each iteration, the vectors, a and c, are updated.
4. At the end of the iterations, the position of the alpha wolf is presented as the “optimal point”.

Integrating the GWO with ANN, assures that the GWO algorithm considers the combinations of bias and weights, as the cost function and optimizes the result ro reach the maximum efficiency [35].

#### 2.3.3. ANFIS

**Figure 2:**
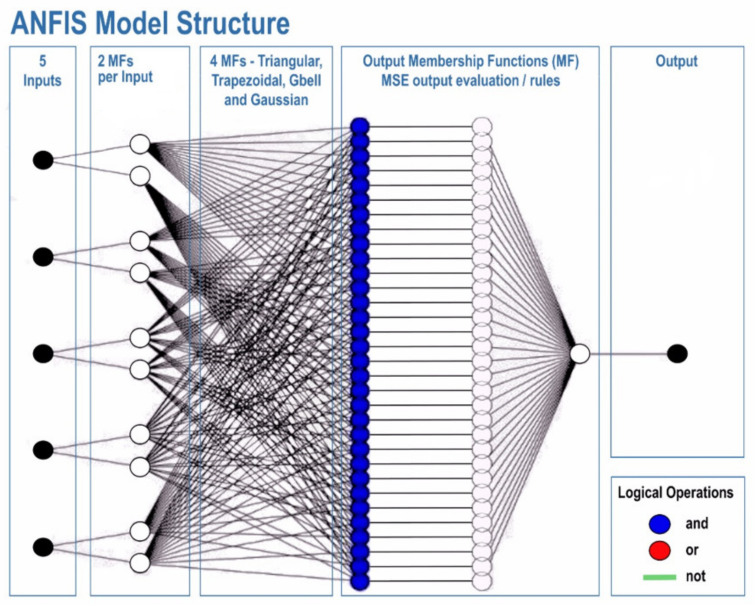
The architecture of the adaptive neuro fuzzy inference system (ANFIS) model.

The ANFIS modelling system is based on the comparison of values, set of rules, input membership functions, output membership functions, multiple inputs and an output (Figure 2). It is a type of artificial neural system, based on the Takagi-Sugeno fuzzy interference system. The adaptive neuro fuzzy inference system (ANFIS) is used for many hybrid-based data, it combines intelligent technologies to aquire data and produce an relevant output. In other words, an ANFIS is an ANN, integrated by the Takagi–Sugeno fuzzy inference system. This technique was developed in the early 1990s, it has the benefits and advantages of both an ANN and a fuzzy inference system, it is consistent with the if-then fuzzy set of rules, which can be taught to approximate nonlinear functions. Hence, ANFIS has been proposed as a universal estimator. A more detailed description of ANFIS models, in terms of mathematical models, is available, in our recently developed work [36]. Figure 2, shows the main architecture of the ANFIS model, which is used in the present study.

The training process was initiated by five inputs, using 80% of total data. Two MFs were considered for each input. The training was performed for four different types of MFs, including, triangular, trapezoidal, Gbell and Gaussian MFs. In each training, the output values were compared by mean square error (MSE), as the evaluation criteria, for calculating the accuracy of the developed model. Each training process was performed during epoch number 500. The lowest MSE refers to the best prediction model. After finding the best, the testing process was performed in the presence of the rest of the data (20%).

### 2.4. Evaluation Criteria

The evaluation process is a step for calculating the accuracy of model, for finding the best solution, for the related prediction task. In the present study, the two most frequently used evaluation criteria are mean absolute error (MAE), mean error (ME) and root mean square error (RMSE). These functions employ the output and target values, for calculating their distances. The following are the MAE and RMSE equations [37,38]:

(12) $$\mathrm{MAE}=\frac{\sum_{i=1}^{n}\left|x-y\right|}{n}$$

(13) $$\mathrm{ME}=\frac{\left(x-y\right)}{n}$$

(14) $$\mathrm{RMSE}=\sqrt{\frac{\sum_{i=1}^{n}{\left(x-y\right)}^{2}}{n}}$$

where, in Equations (12)–(14), *x* and *y* are the target and predicted values, respectively, and *n,* refers to the total number of data points.

## 3. Results

This section presents the statistical analysis of the dataset employed for the modeling section. Accordingly, the dataset has been analyzed using the analysis of variance (ANOVA) test by SPSS software. Table 2 presents the sum of squares, df value, mean square, F and significance index for the relation of the target variable with each independent variables by three criterions containing combined, linearity and deviation from linearity. As is clear from Table 2, the effects of all independent variables on the target value are significant and benefit from high linearity value. In fact, this analysis is done for the initial examination of the selection of independent variables for the modeling process.

### 3.1. Training Results 

Table 3 presents the results for MLP model. MLP was compared in the term of number of neurons in the hidden layer. As is clear, MLP, with 20 neurons in the hidden layer, provided the best performance (lower MAE, RMSE and ME as 0.283239, 0.167089 and 0.0751, respectively) compared with others. In addition, MLP architecture with 20 neurons in the hidden layer will be employed for the development of the hybrid MLP-GWO algorithm. 

Table 4 presents the results for the training phase of ANFIS model. Four main MF types including triangular, trapezoidal, Gbell and Gaussian MF types were employed for developing the ANFIS in a training phase, with two MFs and optimum method type hybrid, with an output MF type—linear. The Gaussian MF type with a lower MAE, RMSE and ME (0.251187, 0.025520 and 0.0745, respectively) was selected as the best MF type for developing the ANFIS model. 

MLP with 20 neurons in the hidden layer (selected from the last step), was selected to be integrated by GWO. Table 5 presents the training results for the employed MLP-GWO. The models differ in the number of populations. The number of population 300, was selected as the optimum number of the population having a lower MAE, ME and RMSE (0.247638, 0.088364 and 0.0718, respectively), compared with other treatments.

### 3.2. Testing Results

Table 6 compares the testing results of the selected models, from the training stages. As is clear, MLP-GWO followed by ANFIS provided the lower MAE, ME and RMSE values (0.077281, 0.114355 and 0.3328).

**Figure 3:**
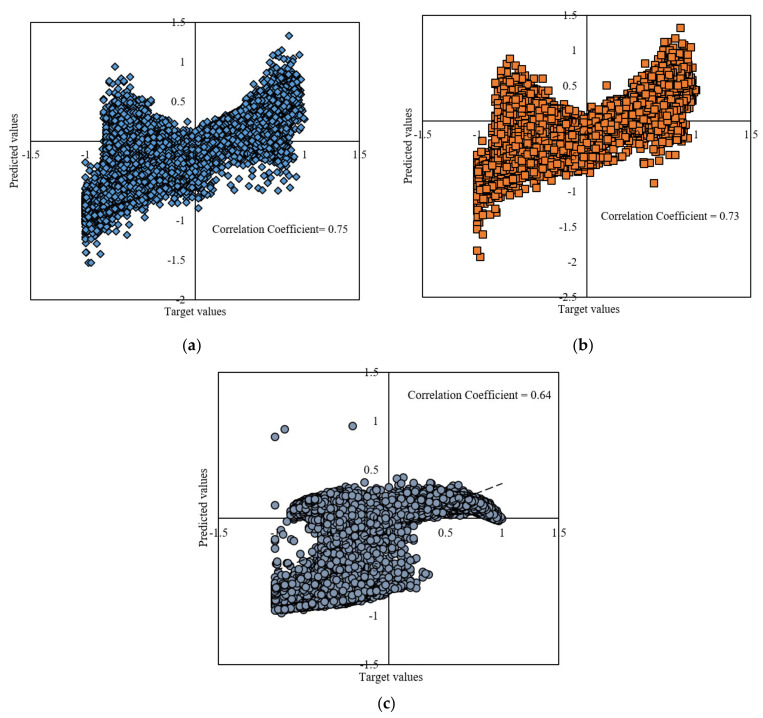
Plot diagram for multi-layer perceptron grey wolf optimizer (MLP-GWO) (**a**), ANFIS (**b**) and MLP (**c**).

The results are shown graphically in Figure 3. The illustration is done through individual and collective models’ representation. 

**Figure 4:**
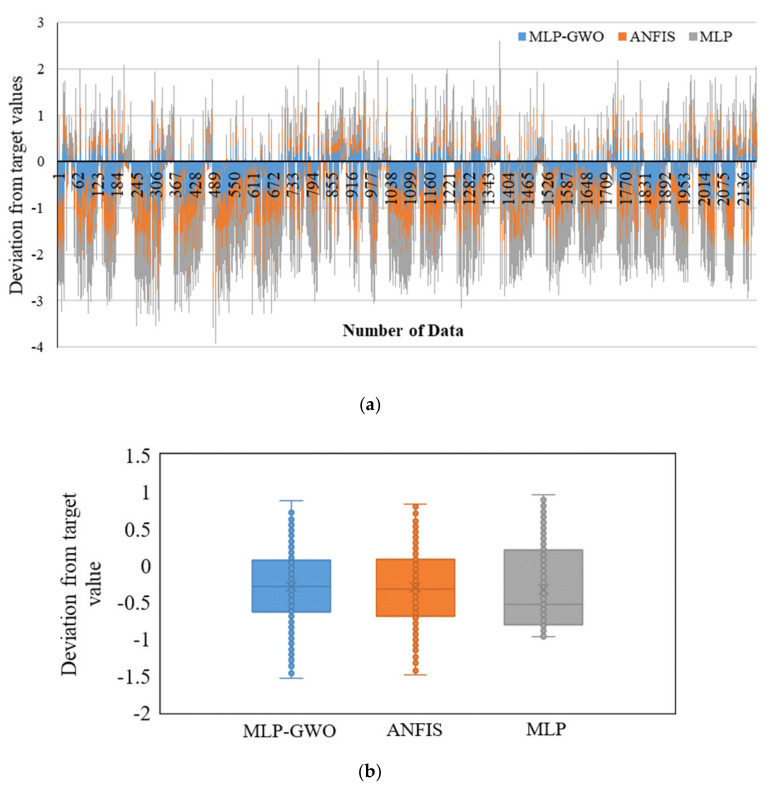
Deviations from target values for all models (**a**) deviation from target values vs. number of data; (**b**) boxplot for deviation from target values for MLP-GWO, ANFIS, and MLP.

Figure 3 presents the scattering results of the testing phase in the presence of predicted and target values. According to Figure 3, the dispersion of target values against predicted values for MLP-GWO is lower than that for the ANFIS and MLP. The lower dispersion refers to the higher accuracy and lower error between target and predicted values and shows that the predicted and target values are close to each other, and the model could successfully predict the target values. Figure 4a,b also presents the deviation from target values. As is clear, the lowest deviation from target values is related to the MLP-GWO. This clearly shows that MLP-GWO could successfully overtake the ANFIS and MLP in the term of prediction accuracy. This also confirms the claims of Figure 3. This can be due to the effect of GWO on the proper optimizing and adopting the weights and bias values of the MLP for generating the output values with a high accuracy. On the other hand, GWO helps the model to overcome the drawback of standard of the MLP.

## 4. Discussion

Currently, there is important work being done in the area of diffuse irradiance and diffuse fraction data collection and prediction. This irradiance information is significant in the planning and efficient implementation of buildings, energy power systems and almost all agricultural applications. For instance, it has been shown that the diffuse fraction irradiance can impact a buildings cooling by 2.3 to 5.18% in Taipei, Taiwan [39,40,41]. The accurate estimation of diffuse irradiance, on a horizontal surface, is highlighted by recent findings of poorly calculated diffuse irradiance values being off by as much as ±8%, for the annual energy yield of photovoltaic systems [42]. Accurate raw data has been, and is currently being, remotely collected via satellite systems. The European Organization for the Exploitation of Meteorological Satellites (EUMETSAT) Satellite Application Facility for Land Surface Analysis (LSA SAF) has been providing “near real-time” estimates of surface radiation data, since 2005 and recent work provides diffuse fraction data, every 15 min for the satellite coverage areas of Europe, Africa, the Middle East, and parts of South America [43]. 

In this paper, three current machine learning models are trained and evaluated for the prediction of the diffuse solar fraction, using recorded data from Almeria, Spain. Diffuse fraction models are highly sensitive to local meteorological conditions and are currently not transferable to disparate localities. The diffuse data used for this work is from an area of the world that experiences a high frequency of cloudless days and enjoys a high-level solar economy, it is, therefore, more predictable in nature, owing to a high clearness index. One study, from Vienna [44], evaluated eight different diffuse fraction models and found that the top three models, using data from Vienna, produced a relative error of less than ±20%. The performance for the top three models in Vienna was very close, showing only a slight, two percent improvement after model calibration. Using hybrid machine learning and artificial intelligence algorithms, there appears to be room for prediction improvements in the future. MLP-GWO has owned the highest performance compared with ANFIS and single MLP. In fact, GWO is considered as a meta-heuristic optimization method. The main points of a meta-heuristic method are simplicity and their flexibility for solving different problems. In addition, a meta-heuristic method has derivation-free mechanisms which enables them to optimize problems stochastically. On the other hand, the superior of a meta-heuristic method is its ability to avoid local optima compared to conventional optimization techniques [45,46]. All these advantages can be considered as strengths of the GWO based hybrid models and increase the accuracy of the network. Therefore, all the above mentioned advantages help MLP-GWO to be superior compared with MLP and ANFIS. Such hybridized machine learning models have shown promising results and it is expected to increase popularity in solar energy applications due to higher performance. 

## 5. Conclusions

In the present study, three robust ML models, a MLP, an ANFIS, and a hybrid MLP-GWO, are advanced for the prediction of the diffuse fraction of solar irradiance for Almeria, Spain. Results were evaluated using two frequently used evaluation criteria, including MAE and RMSE. According to the results, MLP-GWO followed by ANFIS provided higher performance in both the training and the testing stages. MLP-GWO outperformed other models where MAE, ME and RMSE are reported 0.077281, 0.3328 and 0.114355 for testing, respectively. For future research, the use of more sophisticated hybrid machine learning models is suggested. Hybridization for the training of machine learning models shows significant improvement in the performance and accuracy of the models. Therefore, future models can significantly benefit from novel evolutionary algorithms and nature-inspired optimization methods, used to better tune the parameters of the machine learning models, as well as, explore their algorithmic impact on the quality control of a given dataset. Furthermore, the comparative analysis of an standard artificial neural networks, a neuro-fuzzy and a hybrid model revealed the applicability of hybridized models in modeling diffuse fraction. For the future research, implementation of several new comparative analysis is strongly encouraged to investigate the potential of other machine learning models, in articular hybrid and ensemble models.

## Figures and Tables

**Table 1:** Inputs and Output.

Input Data	Output
Global Irradiance (W/m^2^) Beam Irradiance (W/m^2^) Sunshine Duration Index k_t_ (Global/Extraterrestrial-Clearance Index) k (Diffuse/Extraterrestrial)	k_d_ (Global/Diffuse-Diffuse Fraction)

**Table 2:** Results of the analysis of variance (ANOVA) test.

Parameters		Sum of Squares	df	Mean Square	F	Sig.
Global Irradiance*k_d_	(Combined)	362.670	6261	0.058	2.423	0.000
	Linearity	158.377	1	158.377	6624.567	0.000
	Deviation from Linearity	204.292	6260	0.033	1.365	0.000
Beam Irradiance*k_d_	(Combined)	342.804	6073	0.056	2.083	0.000
	Linearity	190.532	1	190.532	7031.608	0.000
	Deviation from Linearity	152.272	6072	0.025	0.925	0.998
Sunshine Duration Index*k_d_	(Combined)	174.180	20	8.709	316.456	0.000
	Linearity	154.287	1	154.287	5606.259	0.000
	Deviation from Linearity	19.893	19	1.047	38.045	0.000
k_t_*k_d_	(Combined)	374.097	776	0.482	49.110	0.000
	Linearity	194.204	1	194.204	19,783.581	0.000
	Deviation from Linearity	179.894	775	0.232	23.646	0.000
k*k_d_	(Combined)	374.097	776	0.482	49.110	0.000
	Linearity	194.204	1	194.204	19,783.581	0.000
	Deviation from Linearity	179.894	775	0.232	23.646	0.000

**Table 3:** Training results related to MLP.

No. of Neurons in the Hidden Layer	MAE	RMSE	ME
15	0.329652	0.381277	0.0860
20	0.283239	0.167089	0.0751
25	0.303247	0.160102	0.0934
30	0.294706	0.187014	0.0886

**Table 4:** Training results related to ANFIS.

Description	MF Type	MAE	RMSE	ME
No. of MFs = 2 Optimum method = hybrid Output MF type = linear	Triangular	0.252980	0.341010	0.0749
	Trapezoidal	0.267428	0.096249	0.0768
	Gbell	0.253935	0.089634	0.0748
	Gaussian	0.251187	0.025520	0.0745

**Table 5:** Training results related to MLP-GWO.

No. of Population	MAE	RMSE	ME
100	0.262107	0.343945	0.0733
200	0.253794	0.093941	0.0786
300	0.247638	0.088364	0.0718
400	0.25512	0.097463	0.0736

**Table 6:** Testing results for the selected models.

Model Name	MAE	RMSE	ME
MLP	0.503710	0.550427	0.4589
ANFIS	0.422157	0.516688	0.4392
MLP-GWO	0.077281	0.114355	0.3328

## Nomenclatures

DF	Diffuse Fraction
MLP	Multi-Layered Perceptron
ML	Machine Learning
ANFIS	Adaptive Network-based Fuzzy Inference System
GWO	Grey Wolf Optimizer
RMSE	Root Mean Square Error
MAE	Mean Absolute Error
LUE	Light Use Efficiency
IEA	International Energy Agency
PV	Photo Voltaic
ANN	Artificial Neural Network
MSE	Mean Square Error
MF	Membership Function
ANOVA	Analysis of Variance
NREL	National Renewable Energy Laboratory
